# A comparative assessment of myelin-sensitive measures in multiple sclerosis patients and healthy subjects

**DOI:** 10.1016/j.nicl.2022.103177

**Published:** 2022-08-30

**Authors:** Reza Rahmanzadeh, Matthias Weigel, Po-Jui Lu, Lester Melie-Garcia, Thanh D. Nguyen, Alessandro Cagol, Francesco La Rosa, Muhamed Barakovic, Antoine Lutti, Yi Wang, Meritxell Bach Cuadra, Ernst-Wilhelm Radue, Laura Gaetano, Ludwig Kappos, Jens Kuhle, Stefano Magon, Cristina Granziera

**Affiliations:** a Translational Imaging in Neurology Basel, Department of Medicine and Biomedical Engineering, University Hospital Basel and University of Basel, Basel, Switzerland; b Neurologic Clinic and Policlinic, MS Center and Research Center for Clinical Neuroimmunology and Neuroscience Basel (RC2NB), University Hospital Basel and University of Basel, Basel, Switzerland; c Division of Radiological Physics, Department of Radiology, University Hospital Basel, Basel, Switzerland; d Department of Radiology, Weill Cornell Medical College, New York, NY, USA; e Signal Processing Laboratory (LTS5), Ecole Polytechnique Fédérale de Lausanne (EPFL), Lausanne, Switzerland; f CIBM Center for Biomedical Imaging, Lausanne, Switzerland; g Radiology Department, Lausanne University and University Hospital, Lausanne, Switzerland; h Laboratory for Research in Neuroimaging, Department of Clinical Neurosciences, Lausanne University Hospital and University of Lausanne, Lausanne, Switzerland; i F. Hoffmann-La Roche Ltd., Basel, Switzerland; j Pharmaceutical Research and Early Development, Roche Innovation Center Basel, F. Hoffmann-La Roche Ltd., Basel, Switzerland

**Keywords:** Multiple sclerosis, Quantitative MRI, Myelin, Reproducibility, Sensitivity

## Abstract

**Introduction:**

Multiple Sclerosis (MS) is a common neurological disease primarily characterized by myelin damage in lesions and in normal - appearing white and gray matter (NAWM, NAGM).

Several quantitative MRI (qMRI) methods are sensitive to myelin characteristics by measuring specific tissue biophysical properties. However, there are currently few studies assessing the relative reproducibility and sensitivity of qMRI measures to MS pathology *in vivo* in patients.

**Methods:**

We performed two studies. The first study assessed of the sensitivity of qMRI measures to MS pathology: in this work, we recruited 150 MS and 100 healthy subjects, who underwent brain MRI at 3 T including quantitative T1 mapping (qT1), quantitative susceptibility mapping (QSM), magnetization transfer saturation imaging (MTsat) and myelin water imaging for myelin water fraction (MWF). The sensitivity of qMRIs to MS focal pathology (MS lesions vs peri-plaque white/gray matter (PPWM/PPGM)) was studied lesion-wise; the sensitivity to diffuse normal appearing (NA) pathology was measured using voxel-wise threshold-free cluster enhancement (TFCE) in NAWM and vertex-wise inflated cortex analysis in NAGM. Furthermore, the sensitivity of qMRI to the identification of lesion tissue was investigated using a voxel-wise logistic regression analysis to distinguish MS lesion and PP voxels.

The second study assessed the reproducibility of myelin-sensitive qMRI measures in a single scanner. To evaluate the intra-session and inter-session reproducibility of qMRI measures, we have investigated 10 healthy subjects, who underwent two brain 3 T MRIs within the same day (without repositioning), and one after 1-week interval. Five region of interest (ROIs) in white and deep grey matter areas were segmented, and inter- and intra- session reproducibility was studied using the intra-class correlation coefficient (ICC). Further, we also investigated the voxel-wise reproducibility of qMRI measures in NAWM and NAGM.

**Results:**

qT1 and QSM showed the highest sensitivity to distinguish MS focal WM and cortical pathology from peri-plaque WM (P < 0.0001), although QSM also showed the highest variance when applied to lesions. MWF and MTsat exhibited the highest sensitivity to NAWM pathology (P < 0.01). On the other hand, qT1 appeared to be the most sensitive measure to NAGM pathology (P < 0.01). All myelin-sensitive qMRI measures exhibited high inter/intra sessional ICCs in various WM and deep GM ROIs, in NAWM and in NAGM (ICC 0.82 ± 0.12).

**Conclusion:**

This work shows that the applied qT1, MWF, MTsat and QSM are highly reproducible and exhibit differential sensitivity to focal and diffuse WM and GM pathology in MS patients.

## Highlights

• qT1 and QSM showed the highest sensitivity to distinguish MS focal WM and cortical pathology from peri-plaque.
• MWF and MTsat exhibited the highest sensitivity to NAWM pathology.
• qT1 appeared to be the most sensitive measure to NAGM pathology.
• All myelin-sensitive qMRI measures exhibited high intra-scanner reproducibility.

## 1 Introduction

In the last decades, several myelin-sensitive quantitative magnetic resonance imaging (qMRI) techniques (e.g. myelin water imaging, magnetization transfer, T1 relaxometry, quantitative susceptibility imaging) have been developed, which provide more specific measures of demyelination and remyelination in multiple sclerosis (MS) patients than conventional MRI (Piredda et al., 2021, Granziera et al., 2021). To date, however, it is unclear how all these measures compare to each other, both in terms of sensitivity to MS damage and of reproducibility across different acquisition sessions (van der Weijden et al., 2021).

MS lesions contain variable amounts of inflammatory infiltrates, demyelination and axonal loss (Lucchinetti et al., 2000). Also, the *peri*-plaque (PP) tissue around the MS lesion is not healthy and contains both myelin and axonal damage although to a lesser degree than the lesion itself (Lieury et al., 2014). Furthermore, the normal-appearing tissue (i.e. white and gray matter areas that do not exhibit signs of focal pathology, NAWM and NAGM, respectively) is characterized by diffuse myelin and axonal damage and microglia clusters (Multiple and Pathology, 2018, Granberg et al., 2017, Cui et al., 2017, Kutzelnigg et al., 2005). To date it is unclear which qMRI measure is the most sensitive to these different pathological hallmarks of MS pathology.

Myelin-sensitive techniques exploit different contrast mechanisms to assess specific characteristics of the myelin sheats. Myelin water imaging (MWI) quantifies the water between myelin layers by distinguishing multiple water components in T2 relaxometry data, and provides measures (e.g. myelin water fraction, MWF) that have been validated postmortem (Moore et al., 2000, Kozlowski et al., 2014). Quantitative susceptibility mapping (QSM) quantifies the spatial distribution of magnetic susceptibility in biological tissue (Liu et al., 2012) and has been shown to be sensitive to iron content and to myelin integrity (Hametner et al., 2018, Wisnieff et al., 2015). Quantitative T1 mapping (qT1) quantifies T1 relaxation times that are sensitive to water content and macro/micro molecules changes within a tissue, such as the one provoked by demyelination and axonal loss in the brain (Granziera et al., 2021, Kolb et al., 2021). Although it is challenging to disentangle the contribution of these factors in acquired qT1, it has been shown that qT1 correlates well with myelin content in NAWM and MS lesions (Mottershead et al., 2003, Seewann et al., 2009). Magnetization transfer (MT) imaging measures the magnetization exchange between protons in free water and protons bound to macromolecules, which has been related to myelin integrity and content (Moccia et al., 2020). MT saturation (MTsat) was developed to improve the MT ratio (MTR) by decoupling the MTR from T1 relaxation time contributions and by incorporating excitation flip angle inhomogeneity, thereby overcoming some limitations of previous MT-based methodologies (Helms et al., 2008). Combined MRI – neuropathology studies have shown that the MT ratio correlates to myelin content within the brain tissue but also to the presence of macrophages and astrocytes (Moccia et al., 2020, Schmierer et al., 2004).

All the above mentioned myelin-sensitive qMRI techniques might provide differential sensitivity to MS pathology hallmarks. Nevertheless, only few previous studies explored the relative sensitivity of some of these measures (e.g., qT1, quantitative T2 and MTR to MS damage in WM (O'Muircheartaigh et al., 2019, Vavasour et al., 2006), but none included QSM and MTsat as well as an assessment of cortical pathology.

Last, most previous studies evaluated myelin-sensitive qMRI reproducibility using single-contrast approaches, which renders it challenging to compare obtained results among different qMRI measures, due to the heterogeneous experimental settings.

In this work, we further expanded previous knowledge about myelin-sensitive qMRI measures by comprehensively evaluating (i) their sensitivity to MS pathology in a large cohort of patients and healthy controls; as well as (ii) their mono-centric reproducibility.

## 2 Materials and methods

### 2.1 Participants

#### 2.1.1 Sensitivity study

To assess sensitivity to MS pathology, we have considered both sensitivity to focal damage (i.e. sensitivity of qMRI to alterations in MS lesions vs periplaque tissue) as well as to diffuse damage (i.e. sensitivity of qMRI to alterations in NAWM and NAGM vs healthy WM and GM). We enrolled 150 MS patients (92 RRMS and 58 PMS) and 100 healthy controls. The inclusion criteria were: (i) MS diagnosis according to McDonald criteria 2018 (Thompson et al., 2018) and diagnosis of active RRMS or inactive PMS as defined by Lublin et al. (Lublin et al., 2014); (ii) absence of any concomitant psychiatric or neurological disease (excluding headache); (iii) absence of contraindication to MRI. The ethical review committee of the University Hospital Basel (IRB of Northwest Switzerland) approved the study, and all participants entered the study following written consent. All subjects underwent MRI at 3 T. Clinical characteristics of patients and healthy controls are reported in Table 1.

**Table 1:** Clinical and demographic characteristics of patients and healthy subjects.

	Multiple sclerosis patients	Healthy subjects
Sex, n (male/female)	150 (58/92)	100 (46/54)
Age (years), mean ± SD	45 ± 15	38 ± 13
EDSS score, median (range)	3.63 (0–8)	–
Disease course (RR/PMS)	92/58	–
Disease-modifying therapy (n)	Untreated (15) Interferon-beta (8) Glatiramer acetate (7) Dimethyl fumarate (26) Fingolimod (16) Natalizumab (3) Rituximab (5) Ocrelizumab (60) Siponimod (2) Teriflunomide (8)	

#### 2.1.2 Reproducibility study

We have enrolled 17 healthy subjects who underwent two scans without repositioning (time point 1 and 2, TP1 and TP2) and a third scan (TP3) was performed 1 week (±3 days) later.

**Fig. 1:**
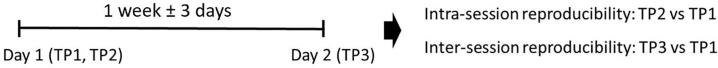
Reproducibility study design. Time points (TP) 1 & 2 were performed without repositioning and TP3 1 week ± 3 days later.

The design of this reproducibility assessment allowed to investigate (i) intra-session reproducibility without subjects repositioning; as well as (ii) inter-session reproducibility after repositioning, Fig. 1.

Seven subjects were excluded from the study: motion artefacts (n = 4), which were to be mainly attributed to the applied study design (scan-rescan without repositioning, acquisition time: 1 h:25 min:12 s), technical issues of the MR system leading to either heavy artifacts (n = 1) r impossibility to acquire MRI in time at TP3 (n = 2) (Fig. 1).

### 2.2 MR acquisition and qMRI maps reconstruction

#### 2.2.1 MR acquisition

MRI was performed on a 3 T whole-body MR system (Magnetom Prisma, Siemens Healthcare, Erlangen, Germany) using a 64-channel phased-array head and neck coil for radio frequency reception.

The MRI protocols included: (i) 3D FLAIR (TR/TE/TI = 5000/386/1800 ms; R = 2 (GRAPPA), 24 integrated reference lines, scan time = 5:40 min) and MP2RAGE (TR/TI1/TI2 = 5000/700/2500 ms; R = 3 (GRAPPA), 32 integrated reference lines, scan time = 8:20 min) both with 1 mm^3^ isotropic spatial resolution; (ii) Fast Acquisition with Spiral Trajectory and adiabatic T2prep (FAST-T2) (TR/TE = 7.5/0.5 ms, six T2prep times = 0 (T2prep turned off), 7.5, 17.5, 67.5, 147.5, 307.5 ms, voxel size = 1.25x1.25x5 mm^3^, scan time = 4.5 min, as described in (Nguyen et al., 2016); as well as (iii) 3D segmented *EPI* with submillimeter isotropic resolution (TR/TE/resolution = 64 ms/35 ms/0.67x0.67x0.67 mm^3^) (Sati et al., 2012). Quantitative Magnetization Transfer saturation (MTsat) images were acquired using three 3D RF spoiled gradient echo acquisitions with predominantly Magnetization Transfer-weighted (MTw: TR/α = 25 ms/5°), proton density-weighted (PDw: TR/α = 25 ms/5°) and T1-weighted (T1w: TR/α = 11 ms/15°) contrast (Helms et al., 2008, Helms and Dechent, 2009, Helms et al., 2009) (Helms et al., 2009, 2008a, 2008b). The MT contrast was achieved by use of a Gaussian-shaped RF pulse prior to the excitation (12.8 ms duration, 520° nominal flip angle, 2.2 kHz frequency offset from water resonance). A single gradient echo was acquired with echo time TE = 4.92 ms. The image resolution was 1.33 mm^3^ isotropic. Parallel imaging was used along the phase-encoding direction (MTw, PDw, T1w: each R = 2 (GRAPPA), 24 integrated reference lines (Griswold et al., 2002), 6/8 partial Fourier was used in both phase-encoding directions. The acquisition times were 1:22 min (T1w) and 3:07 min (MTw, PDw). Data were acquired to calculate radio frequency (RF) transmit field B1^+^ maps using the steady state free precession based B1-TRAP approach (Ganter et al., 2013); and to correct for effects of radio frequency transmit inhomogeneity on the quantitative maps (Helms and Dechent, 2009, Helms et al., 2009). The image resolution of the B1-mapping data was 4 × 4 × 5 mm^3^, echo time TE = 1.76 ms, TR = 2300 ms and flip angle α = 60°. The acquisition time of the B1 mapping sequence was 2:09 min. The total acquisition time for the MTsat protocol was 9:45 min.

#### 2.2.2 qMRI maps reconstruction

T1maps were computed from acquired MP2RAGE data as in (Marques et al., 2010): Based on the two acquired inversion images, MP2RAGE allows reconstructing a so called uniform intensity image that is insensitive to radio frequency reception field (B1^-^) bias, proton density and T2* contrast (Marques et al., 2010). Its sensitivity to the radio frequency excitation field B1^+^ is reduced (Marques et al., 2010). By building a lookup table for these uniform intensities with protocol parameters as dependents and T1 as a free parameter, quantitative T1 maps can be reconstructed (Marques et al., 2010).

MWF maps were reconstructed through the consideration of TE sampling and spatially constrained nonlinear fitting on FAST-T2 data as in (Nguyen et al., 2012). This approach was shown to produce MWF values in the brain that are comparable with those obtained using a multi-echo spin-echo sequence that has been validated against histological myelin measurements (Laule et al., 2006).

Based on the 3D *EPI* sequence that provides a fast whole-brain acquisition with sub-millimeter isotropic spatial resolution, QSM maps were reconstructed by (i) unwrapping the phase of the *EPI* data with the path finding and the SNR as the image-quality guidance (Liu et al., 2012) in the region growth method, (ii) removing the background field through the Projection onto Dipole Fields algorithm, and (iii) using the morphology-enabled dipole inversion algorithm to compute the susceptibility from the local field (MEDI reconstruction), as in Liu *et al.* (Liu et al., 2012)*.* Previous studies have shown that *EPI*-QSM provided similar mean susceptibility values to standard multi-echo GRE-QSM (Wicaksono et al., 2021, Sun et al., 2017).

**Fig. 2:**
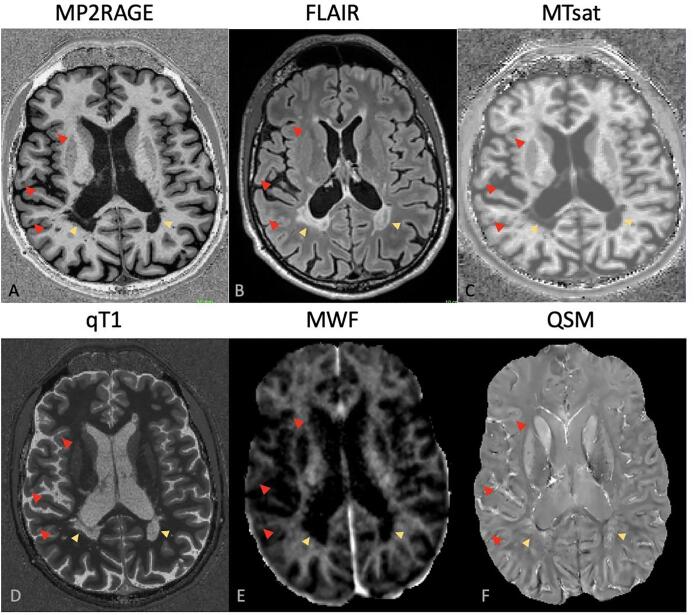
Analysis pipeline for sensitivity study. FLAIR: Fluid-attenuated inversion recovery, WML: white matter lesion, CL: cortical lesion, NAWM: normal-appearing white matter, NAGM: normal appearing gray matter, FS: FreeSurfer, GLM: general linear model, TFCE: threshold-free cluster enhancement.

MTsat maps were calculated from the acquired data by using the hMRI Toolbox (https://github.com/hMRI-group/hMRI-toolbox) (Kaunzner et al., 2019, Tabelow et al., 2019) running under SPM12 (Wellcome Trust Centre for Neuroimaging, London, UK; http://www.fil.ion.ucl.ac.uk/spm) and Matlab 9.9 (R2020b) (Mathworks, Natick, MA, USA). The MTsat maps were computed as described in Helms, Dathe, and Dechent (Helms et al., 2008) and Helms and Dechent (Helms and Dechent, 2009) using the MTw, PDw, and T1w images (Fig. 2).

### 2.3 Lesion/ROIs identification and segmentation

**Fig. 3:**
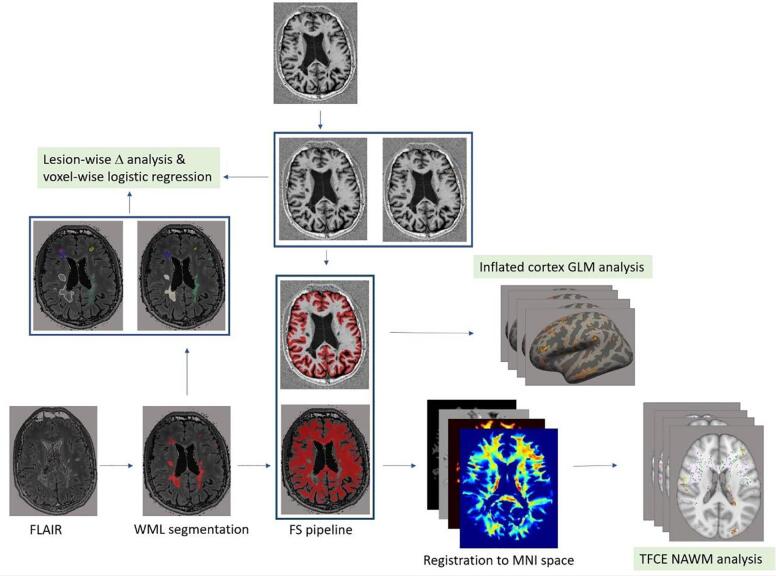
White matter lesions (yellow arrow) and cortical lesios (red arrow) in axial slices obtained from A) MP2RAGE, B) FLAIR, C) MTsat, D) qT1, E) MWF & F) QSM. (For interpretation of the references to colour in this figure legend, the reader is referred to the web version of this article.)

For the sensitivity study, an automatic segmentation of WM lesions was performed by using a publicly available deep learning-based method (La Rosa et al., 2020). This approach consists of a single convolutional neural network and was adapted to take FLAIR and MP2RAGE MRI contrasts as input. Manual correction of automatic WM (n = 4629; lesion size (Median (25 % quartile, 75 % quartile) 16 (Lieury et al., 2014, Nguyen et al., 2012) and cortical lesions (n = 719; lesion size (Median (25 % quartile, 75 % quartile) 53 (Thompson et al., 2018) was performed on FLAIR and MP2RAGE images by consensus (WM: RR and CG, cortical: RR and AC) (Geurts et al., 2011). Both intracortical lesions and the GM part of leukocortical lesions were considered as “cortical lesions”. To identify the GM part of leukocortical lesions, we first manually segmented both their GM and WM part, and then masked them with the GM mask obtained with FreeSurfer (Rahmanzadeh et al., 2021). A few patients (n = 3) with dirty WM were excluded from study because for those accurate delineation of lesion border was not possible, which could have led to inaccurate estimation of qMR in lesions, PP and NA tissue (Fig. 3).

For the reproducibility study, six regions of interests (splenium of corpus callosum, genu of corpus callosum, putamen, head of caudate, thalamus and cortical grey matter) segmented automatically by imaging software package FreeSurfer (FS) (v.6.0, surfer.nmr.mgh.harvard.edu) (Fischl, 2012) were manually corrected on FLAIR and MP2RAGE. The ROIs mask were then registered to qMR spaces using a linear registration in FMRIB Software Library (FSL) (Jenkinson et al., 2012) with nearest-neighbor interpolation.

### 2.4 WM and cortex segmentation

For both studies, we used the imaging software package Freesurfer (https://surfer.nmr.mgh.harvard.edu/) to segment the brain into whole WM, cortex, deep grey matter structures, and ventricles. NAWM and NAGM masks were obtained by subtracting WM and cortical lesion masks from WM and cortical masks.

An in-house developed algorithm was used to automatically produce a 2-voxel layer of NAWM surrounding the lesions; herein after denoted *peri*-plaque (PP).

We have opted to assess differences between lesions and *peri*-plaque white matter/grey matter (PPWM, PPGM) to avoid spurious changes due to the underlying anatomy. In fact, all qMRI measures applied in this study are sensitive to the organization of the WM tissue and its microscopic anatomical features (i.e. bundles orientations, myelin content, cellular distribution etc.). Comparing lesions to ROIs in more remote white matter than PPWM will suffer from the bias due to the different tissue microstructure. Furthermore, we have chosen to report absolute values to allow the comparability of the sensitivity of the different qMRI maps to MS pathology. In fact, whilst qT1, MTsat and MWF are expected to respectively increase (qT1) or decrease (MTsat and MWF) based on previous literature (summarized in Granziera et al. (Granziera et al., 2021), QSM may either increase (due to myelin loss or iron accumulation) or decrease (due to remyelination or edema accumulation) or both (Chen et al., 2014).

The absolute delta of changes between lesions and periplaque tissue was calculated as follows: (Δ^WML-PPWM^ & Δ^CL-PPGM^) between lesion and PP tissue for WMLs and CLs as follows:

Δ^lesion-PP^: (mean qMRI measure in lesion – in PP) / mean qMRI measure in PP (formula (1)).

A minority of lesions were excluded from the analysis (353 WMLs (7.6 % of all WMLs) and 217 CLs (30 % of all CLs)), due to the challenges in establishing an automatic procedure to assess the PP-tissue in i) lesions of very small size (due to partial-volume effects) ii) lesions close to ventricle wall and WM-GM border or iii) lesions located in/around WM bundles.

To note, QSM studies in the cortical areas are challenging due to cortical erosion and the presence of banding/streaking artifacts (Yaghmaie et al., 2021). In this work, we minimized these issues by (i) identifying the lowest erosion threshold that did not impact the obtained QSM values; and (ii) by carefully examining the presence of artifacts in the obtained maps.

For the reproducibility study, FS was performed using MP2RAGE acquired at baseline and FS outputs were then registered to the other time-points using the linear registration with nearest-neighbor interpolation.

### 2.5 Voxel-Based analyses in NAWM

NAWM maps were co-registered patient-wise to a reference brain (standard MNI152 space) using an affine registration (FLIRT) followed by non-linear FNIRT registration in FSL (https://fsl.fmrib.ox.ac.uk/fsl/) (Jenkinson et al., 2012). Then, the NAWM maps were wrapped to WM templates using -- applywarp commandline in FSL. As previously performed (Vrenken et al., 2006), in NAWM we excluded voxels that were not present in at least 50 percent of subjects, and (Granziera et al., 2021) filled missing data with the group mean value of those voxels present in group subjects. By using the randomize tool of FSL with Threshold-Free Cluster Enhancement (TFCE), we carried out a voxel-wise comparison of MWF, qT1, MTsat and QSM maps (i) between patients and controls (sensitivity analysis); and (ii) between TP1-TP2 & TP1-TP3 (reproducibility analysis). *P* values<0.01 were considered statistically significant. To compare the extent of qMRI measure changes in areas showing significant clusters, the mean qMRIs maps were computed using FSL.

### 2.6 Vertex-wise analysis in NAGM

A customized volume-to-surface mapping algorithm was applied to voxels assigned to the grey matter ribbon by FreeSurfer - i.e., voxels with coordinates located between the white and pial surfaces were registered and projected into a standard surface. A smoothing kernel of 10-mm full-width at half-maximum was used. Then, generalized linear model (GLM) analysis was conducted to assess (i) the sensitivity of each map in NAGM to assess damage between patients and controls; and (ii) to assess the intra- and inter-scanner reproducibility of each qMRI measure in GM (TP1 vs TP2 & TP1vs TP3, respectively). *P* values<0.01 were considered statistically significant.

### 2.7 Intra-class correlation coefficient to assess intra- and inter-session reproducibility

In six regions of interest (ROIs: genu and splenium of the corpus callosum, putamen, head of caudate, thalamus and cortical GM), we calculated the intra-class correlation coefficient (ICC) for each qMRI measure using a two-way model with consistency of agreement within and across sessions (TP1-TP2, TP1-TP3). The ICC analysis was performed in Stata 16 statistical package. According to Koo et al. (Koo and Li, 2016), ICC < 0.5, 0.5 ≤ ICC < 0.75, 0.75 ≤ ICC < 0.90, 0.90 ≤ ICC were interpreted as indicative of poor, moderate, good and excellent reliability, respectively.

### 2.8 Statistical analyses

Statistical analysis was performed using GraphPad Prism version 8.0.0 for Windows, GraphPad Software, San Diego, California USA. Kolmogorov-Smirnov’s test was used to assess the normality of data. Non-parametric Mann-Whitney test and Kruskal-Wallis test with Dunn’s test for multiple comparisons correction, were used for unpaired two-group analysis and more-than-three group analysis, respectively.

To account for both differences in mean and variance in the comparison among myelin-sensitive measures, we repeated the same comparison for Z-scores of delta values, as follows:

Z score = (lesion Δ - mean lesion Δ across all lesions) / standard deviation of lesion Δ across all lesions (formula (2)).

We have pooled RRMS and PMS together, because there is increasing evidence showing that there’s no difference in the microstructural characteristics of focal pathology between RRMS and PMS (Lucchinetti et al., 2000, Rahmanzadeh et al., 2021, Antel et al., 2012).

For the independent two samples analysis, a Cohen’s d effect size was calculated as Cohen's *d* = (*M*_2_ - *M*_1_) ⁄ *SD*_pooled_, where *M*_1_ and *M*_2_ are the group means and *SD*_pooled_ = √((*SD*_1_^2^ + *SD*_2_^2^) ⁄ 2).

## 3 Results

### 3.1 qMRI measures sensitivity to focal and diffuse MS pathology

#### 3.1.1 Sensitivity of myelin-sensitive qMRI measures to focal MS pathology

**Fig. 4:**
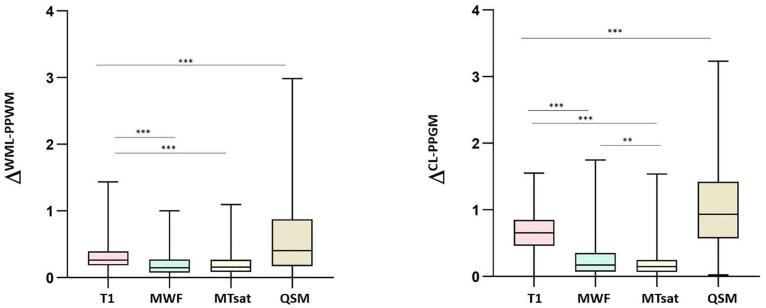
Comparison of the sensitivity of qMRI measures in distinguishing lesion vs *peri*-plaque tissue in WM and in the cortex (because of QSM changes in both direction in MS, absolute Δ^WML-PPWM^ & Δ^CL-PPGM^ are reported). * P < 0.05, ** P < 0.001, *** P < 0.0001.

**Fig. 5:**
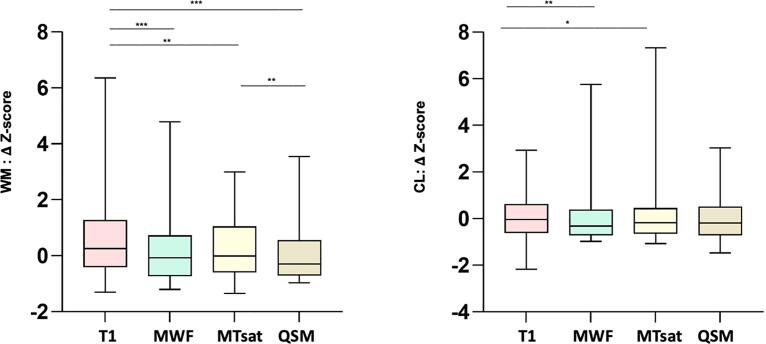
Comparison of Z-score of qMRI delta measures between lesion vs *peri*-plaque tissue in WM and in the cortex. * P < 0.05, ** P < 0.001, *** P < 0.0001.

WM lesion-wise analysis showed that mean Δ^WML-PPWM^ was the highest for QSM and the lowest for MTsat (Fig. 4, Table 2); Δ^WML-PPWM^ was higher for QSM than for qT1 (P < 0.0001; Cohen’s *d* effect size = 0.69) and for qT1 than for MWF (P < 0.0001; Cohen’s *d* effect size = 0.69). Likewise, qT1 shows higher mean Z-score compared to MWF and MTsat (both P < 0.001). However, in contrast to Δ analysis, the mean Z-score for QSM was lower than the one of MWF and qT1 (both P < 0.001; Fig. 5).

**Table 2:** Summary of Mean and standard deviations (M, SD) for Δ^WML-PPWM^ and Δ^CL-PPGM^.

	MWF	qT1	MTsat	QSM
Δ^WML-PPWM^ (M ± SD)	0.19 ± 0.16	0.31 ± 0.17	0.18 ± 0.13	0.64 ± 0.66
Δ^CL-PPGM^ (M ± SD)	0.25 ± 0.26	0.66 ± 0.30	0.18 ± 0.17	1.07 ± 0.71

Cortical lesion-wise analysis also showed that mean Δ^CL-PPGM^ was the highest for QSM and the lowest for MTsat (Fig. 4, Table 2); Δ^CL-PPGM^ was higher for QSM than for qT1 (P < 0.001; Cohen’s *d* effect size = 0.75) and for qT1 than for MWF (P < 0.0001) (Cohen’s *d* effect size = 1.46). When the z-score analysis was considered, qT1 showed the highest mean Z-score compared to MWF and MTsat (both P < 0.05; Fig. 5). However, in contrast to the Δ analysis, the mean Z-score for QSM was not higher than the one measured using the other myelin sensitive-measures.

#### 3.1.2 Sensitivity of qMRI measures to NAWM pathology in MS

**Fig. 6:**
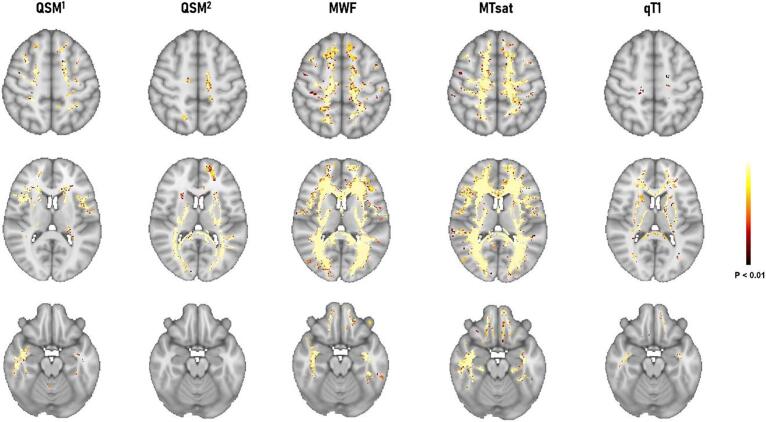
Voxel-wise TFCE comparison between MS patients and healthy subjects in NAWM and vertex-wise inflated cortex analysis in NAGM. QSM^1^ showed areas where susceptibility values are higher in patients vs controls and QSM^2^ shows areas in which susceptibility is lower in patients vs controls.

Voxel-wise analysis showed that all qMRI measures were sensitive to changes in some clusters of NAWM voxels compared to WM in healthy controls. However, MTsat, MWF, QSM and qT1 showed decreasing sensitivity to NAWM pathology in MS (Fig. 6, total clusters voxel number for MWF: 285820, MTsat: 340304, QSM (QSM1 + QSM2): 132548, qT1: 36757; *P* < 0.01).

The average qMRI measures in clusters of significant difference between patients and controls (P < 0.01) were as follows: qT1 = 799.88 vs 768.27 ms (patients vs controls); MWF = 6.35 vs 7.09p.u. (percent unit, patients vs controls); MTsat = 1.39 vs 1.55p.u. (patients vs controls); QSM1 = -12.15 vs −18.77 ppb (patients vs controls); QSM 2 = 26.08 vs −18.39 ppb (patients vs controls) in QSM2.

#### 3.1.3 Sensitivity of qMRI measures to NAGM pathology in MS

**Fig. 7:**
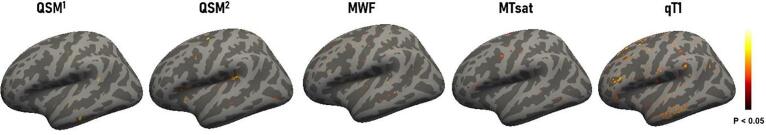
Vertex-wise inflated cortex analysis between MS patients and healthy subjects in NAGM. QSM^1^ shows areas where susceptibility values are higher in patients vs controls and QSM^2^ shows areas in which susceptibility is lower in patients vs controls.

Vertex-wise cortical surface analysis showed that all qMRI measures were sensitive to changes in some clusters of NAGM voxels compared to GM in healthy controls. However, qT1, QSM, MWF and MTsat appeared to show decreasing sensitivity to NAGM pathology in MS (Fig. 7, total clusters voxel number for qT1: 4223, QSM (QSM1 + QSM2): 2678, MWF: 926, MTsat: 876; *P* < 0.01). Except for QSM, for other qMR the surface analysis yielded no significant cluster for the comparison MS > HC.

### 3.2 Reproducibility of myelin-sensitive qMRI measures in a single scanner

#### 3.2.1 ICC analysis

Intra-session (TP1-TP2) and inter-session (TP1-TP3) ICCs for qMRI measures are summarized in Table 3.

**Table 3:** ICCs comparison among myelin-sensitive qMRI measures.

qMRI	Comparison	Splenium	Genu	Putamen	Caudate	Thalamus	Cortical GM
MWF	Intra - session Inter - session	0.94 0.90	0.8 0.82	0.81 0.71	0.89 0.50	0.98 0.88	0.92 0.89
qT1	Intra - session Inter - session	0.86 0.95	0.83 0.92	0.98 0.93	0.92 0.77	0.94 0.84	0.89 0.89
MTsat	Intra - session Inter - session	0.82 0.68	0.79 0.73	0.85 0.77	0.59 0.67	0.93 0.88	0.95 0.88
QSM	Intra - session Inter - session	0.77 0.84	0.57 0.62	0.91 0.81	0.93 0.90	0.91 0.87	0.85 0.92

qT1 exhibited the highest intra-session and inter-session reproducibility (ICCs TP1 vs TP2: 0.83–0.98; ICC TP1 vs TP3: 0.77–0.95, respectively). MWF followed with an intra-session ICC of 0.81–0.98, and an inter-session ICC of 0.50–0.90, with lower ICCs in deep gray matter structures than CC parts (Table 3). Also, MTsat and QSM exhibited relatively high ICC in most ROIs (MTsat: intra-session ICC = 0.59–0.95 and inter-session ICC = 0.67–0.88; QSM: intra-session ICC = 0.57–0.93 and inter-session ICC = 0.62–0.90, Table 3).

#### 3.2.2 Voxel-wise WM reproducibility analysis

For all qMRI measures, WM voxel-wise analysis exhibited no clusters of significant intra- and inter-session differences (*P* < 0.01).

#### 3.2.3 Vertex-wise GM reproducibility analysis

Vertex-wise cortical surface analysis showed very small areas of intra- and inter-session differences for all qMRI measures. The total clusters numbers for each measure were as follows: qT1 = 155, QSM (QSM1 + QSM2) = 182, MWF = 312, MTsat = 297 (*P* < 0.01). There was also no substantial overlap between the significant clusters in reproducibility (TP1 vs TP2) and sensitivity (patients vs controls) as outputs of vertex-wise inflated cortex analyses.

## 4 Discussion

In this work, we performed a comprehensive assessment of the sensitivity of myelin-sensitive qMRI measures to MS pathology, as well as of their respective intra-scanner reproducibility.

Our results showed that qMRI measures such as MWF, MTsat, qT1, and QSM exhibit differential sensitivity to MS pathology in MS plaques and in WM and cortical regions outside areas of focal damage.

When a comparison of the mean changes of qMRI measures in lesion vs periplaque tissue was performed, qT1 and QSM showed the highest sensitivity to MS lesions pathology in both WM and cortical GM. This may be due to the broader sensitivity of qT1 to the tissue destruction in CNS encompassing demyelination, axonal degeneration, edema and tissue destruction (Lucchinetti et al., 2000, Lassmann, 2018), which are predominant within the core of the lesion and present to a smaller extent in the *peri*-plaque tissue (Mustafi et al., 2019, Rahmanzadeh et al., 2021, Vrenken et al., 2006, Rodriguez et al., 1993). The higher sensitivity of qT1 for cortical focal pathology may also – at least in part - depend on the fact that qT1 maps were obtained using the same acquisition where cortical lesions were detected (MP2RAGE). On the other hand, the quantification of MWF, MTsat in cortical lesions may have suffered from partial volume effects due to the lower spatial resolution of the respective images compared to MP2RAGE. Interestingly, when the “sensitivity” to MS pathology was calculated using z-scores instead of mean delta measures, results were very similar with the exception of the ones obtained for QSM, which appeared the “least sensitive” among all. Assessing the sensitivity of a given measure using z-scores instead of the “delta of the mean” has the advantage to consider the variance of that measure, which is an important aspect if that variance is of technical origin (i.e. a higher variance will reduce the sensitivity to pathology). Nevertheless, we recently showed that the variance of QSM measures among MS lesions has an important pathological source (Rahmanzadeh et al., 2021), Therefore, we concluded that that the most appropriate way to show the sensitivity to MS pathology is the one that does not penalize a measure because of its variance (i.e. the mean delta): by using this measure qT1 and QSM appeared to outperform MWF and MTsat to identify focal MS lesion pathology. Interestingly, the sensitivity of qT1 to NAWM pathology was the lowest among all qMRI measures. In contrast, MTsat and MWF were the most sensitive to NAWM pathology. Indeed, MWF and MTsat exploit different contrast mechanisms allowing to detect subtle changes in myelin water and lipid/macromolecular content, which may be the consequence of diffuse microglia activation in NAMW (Cui et al., 2017, Rodriguez et al., 1993, Rodriguez and Scheithauer, 1994).

As to NAGM pathology, qT1 and QSM were the most sensitive measures to assess the consequences of neuroinflammatory and neurodegenerative processes occurring in the cortical ribbon of MS patients (Kutzelnigg et al., 2005, Magliozzi et al., 2007). To note, QSM studies in the cortical areas are challenging due to cortical erosion and the presence of banding/streaking artefacts (Yaghmaie et al., 2021). In this work, we minimized these issues by (i) identifying the lowest erosion threshold that did not impact the obtained QSM values; and (ii) by carefully examining the presence of artifacts in the obtained maps.

All qMRI measures exhibited high intra- and inter-session reproducibility; qT1 obtained with MP2RAGE (Marques et al., 2010) exhibited good-to-excellent ICCs (0.77–0.98) in both WM (i.e. splenium/genu of corpus callosum) and in deep gray matter structures. Similarly, qT1 showed the highest reproducibility in voxel-wise and vertex-wise analyses. It is true that the reproducibility of qT1 measures is highly hardware-, software- and sequence-dependent (Bane et al., 2018); nevertheless, the results obtained in this study by using MP2RAGE confirm the feasibility of reproducible measurements across sessions in a single 3 T scanner, which were previously reported (Marques et al., 2010).

As to MTsat, previous reproducibility studies have shown good to excellent ICC for both inter-scanner and intra-scanner acquisitions, both in single-center and multi-center studies (Barker et al., 2005, Weiskopf et al., 2013, Ropele et al., 2005). This is in contrast with the reproducibility of MTR, which is modest across centers due to its high sensitivity to (i) sequence parameters and setup, (ii) the inhomogeneity of the transmitted RF field, and to (iii) the variability in longitudinal relaxation T1 that may occur in different scanners/centers (Helms et al., 2010). Our results support and extend previous findings obtained with MTsat in monocentric assessments by showing an overall good reproducibility for MT in all ROIs except in the caudate (ICC intra-session: 0.59, inter-session: 0.67). Similarly, the voxel-wise WM and vertex-wise cortical analysis showed almost no intra-/inter-session changes in MTsat, a finding that highlights the robustness of MTsat for myelin imaging in a single-scanner and single-center setting.

Regarding QSM, our results confirm previous reports of high intra-scanner and inter-scanner reproducibility for the applied MEDI reconstruction method (Deh et al., 2019). Interestingly, these results are also supported by a multi-center phantom study where the MEDI QSM reconstruction appeared to be highly reproducible (ICC > 0.99) among different clinical and preclinical scanners (Deh et al., 2019). Nevertheless, high reproducibility in QSM experiments seems not to be limited to the MEDI reconstruction method, as also other have shown high intra-scanner reproducibility (i.e. the L2 regularization QSM) (Lin et al., 2015, Santin et al., 2017, Choi et al., 2019), supporting the knowledge that QSM provide robust qMRI measures for multicentric studies.

Similarly, confirming one previous study (Nguyen et al., 2016), our work shows that the reproducibility of FAST-T2 MWF maps is high when assessed both with a region of interest and with a voxel-wise or surface-wise approach in both WM and cortical GM. Previous works also showed a high reproducibility of myelin water fraction maps obtained with other acquisition methods such as GRASE and mcDESPOT (multi-component Driven Equilibrium Single Pulse Observation of T1 and T2) (Lee et al., 2018, Levy et al., 2018, Meyers et al., 2009), although in this case no voxel-wise and surface-wise comparison was performed.

Notably, the reported reproducibility performance does not only depend on the applied acquisition method, but also on (i) the relative spatial resolution/partial volume effects, which may partially explain the outperformance of qT1 maps in cortical regions; as well as on (ii) the applied reconstruction algorithm (eg. MEDI for QSM). A comprehensive evaluation of these aspects is beyond the scope of the current study, but should be considered in future works.

Our work is unique in that it compares different qMRI measures for both focal and diffuse sensitivity to MS pathology as well as for intra-session and inter-session reproducibility at 3 T MRI. Moreover, our study applies qMR measures derived from sequences acquired in a clinically feasible time (acquisition time ranging from 4.5 min for MWF to 9.45 min for MTsat), providing therefore a reference for future clinical trials or clinical applications of myelin-sensitive qMRI. Extending previous studies (Vavasour et al., 2006), our work evaluated the reproducibility of multiple myelin-sensitive qMRI measures not only in specific regions of interest in WM but also in cortical grey matter and deep grey matter nuclei. Further, our work explored first the sensitivity of (i) a clinically-compatible T1 mapping technique such MP2RAGE; (ii) MTsat, a quantitative measure for MT effects that is less sensitive to B1 inhomogeneity and T1 effects than the previously applied MTR (Vavasour et al., 2006); and of (iii) QSM obtained from a fast 3D-*EPI* based T2* weighted acquisition.

Nonetheless, our study provides evidence of a good reproducibility of all qMRI measures only in a single-center and single-scanner setting: therefore, these results warrant confirmation in a future study where reproducibility among different scanners, vendors and field strengths, is assessed. Moreover, our study was not designed to assess reproducibility of time intervals that are normally applied in longitudinal studies (e.g. > 1 year), therefore future work should assess this aspect.

Another limitation of this study was the high drop-out rate (7/17): just over the half (Lucchinetti et al., 2000) due to motion artifacts derived from the length of the protocol including multiple myelin-sensitive qMRI sequences and intra-session reproducibility assessment without repositioning (cf. Materials and Methods section).

In summary, we demonstrated that the applied qMRI measures provide complementary information about the extent of MS pathology in various brain regions, encompassing both WM and GM areas. Our data suggest that the combination of qT1 and MWF might be ideal for a comprehensive study of myelin-damage in the brain of MS patients in single center settings, due to their complementary and optimal sensitivity to focal and diffuse MS-damage in cortical grey and white matter as well as to their high intra-scanner reproducibility. QSM did not seem to provide advantages when average analyses were performed, although we know that it allows to qualitatively identify MS lesion subtypes (Rahmanzadeh et al., 2021, Chen et al., 2014, Absinta et al., 2018). Last, MTsat might be a good compromise for an all-in-one approach due to its relatively high sensitivity to MS damage and reproducibility. Future studies should further assess the relative correlation of qMRI measures with quantification of tissue properties and pathology in MS brains, as well as replicate the current results in larger cohorts of MS patients in vivo.

## CRediT authorship contribution statement

**Reza Rahmanzadeh:** Methodology, Conceptualization, Data curation, Writing – original draft, Writing – review & editing. **Matthias Weigel:** Methodology. **Po-Jui Lu:** Data curation, Methodology. **Lester Melie-Garcia:** Data curation, Methodology. **Thanh D. Nguyen:** Methodology. **Alessandro Cagol:** Data curation. **Francesco La Rosa:** Methodology. **Muhamed Barakovic:** Methodology. **Antoine Lutti:** Methodology. **Yi Wang:** Methodology. **Meritxell Bach Cuadra:** Methodology. **Ernst-Wilhelm Radue:** Methodology. **Laura Gaetano:** Methodology. **Stefano Magon:** Conceptualization, Methodology, Writing – review & editing. **Cristina Granziera:** Conceptualization, Methodology, Supervision, Writing – review & editing.

## Declaration of Competing Interest

The authors declare that they have no known competing financial interests or personal relationships that could have appeared to influence the work reported in this paper.

## Data Availability

Data will be made available on request.

## References

[b0005] Absinta M., Sati P., Fechner A., Schindler M.K., Nair G., Reich D.S. (2018). Identification of Chronic Active Multiple Sclerosis Lesions on 3T MRI. AJNR Am. J. Neuroradiol..

[b0010] Antel J., Antel S., Caramanos Z., Arnold D.L., Kuhlmann T. (2012). Primary progressive multiple sclerosis: part of the MS disease spectrum or separate disease entity?. Acta Neuropathol..

[b0015] Bane O., Hectors S.J., Wagner M., Arlinghaus L.L., Aryal M.P., Cao Y. (2018). Accuracy, repeatability, and interplatform reproducibility of T1 quantification methods used for DCE-MRI: Results from a multicenter phantom study. Magn. Reson. Med..

[b0020] Barker G.J., Schreiber W.G., Gass A., Ranjeva J.P., Campi A., van Waesberghe J.H. (2005). A standardised method for measuring magnetisation transfer ratio on MR imagers from different manufacturers–the EuroMT sequence. MAGMA..

[b0025] Chen W., Gauthier S.A., Gupta A., Comunale J., Liu T., Wang S. (2014). Quantitative susceptibility mapping of multiple sclerosis lesions at various ages. Radiology.

[b0030] Choi J.Y., Lee J., Nam Y., Lee J., Oh S.H. (2019). Improvement of reproducibility in quantitative susceptibility mapping (QSM) and transverse relaxation rates (R 2 *) after physiological noise correction. J. Magn. Reson. Imaging.

[b0035] Cui Q.L., Khan D., Rone M., Johnson R.M., Lin Y.H. (2017). Sublethal oligodendrocyte injury: A reversible condition in multiple sclerosis?. Ann. Neurol..

[b0040] Deh K., Kawaji K., Bulk M., Van Der Weerd L., Lind E., Spincemaille P. (2019). Multicenter reproducibility of quantitative susceptibility mapping in a gadolinium phantom using MEDI+0 automatic zero referencing. Magn. Reson. Med..

[b0045] Fischl B. (2012). FreeSurfer. Neuroimage..

[b0050] Ganter C., Settles M., Dregely I., Santini F., Scheffler K., Bieri O. (2013). B1+-mapping with the transient phase of unbalanced steady-state free precession. Magn. Reson. Med..

[b0055] Geurts J.J., Roosendaal S.D., Calabrese M., Ciccarelli O., Agosta F., Chard D.T. (2011). Consensus recommendations for MS cortical lesion scoring using double inversion recovery MRI. Neurology..

[b0060] Granberg T., Fan Q., Treaba C.A., Ouellette R., Herranz E., Mangeat G. (2017). In vivo characterization of cortical and white matter neuroaxonal pathology in early multiple sclerosis. Brain..

[b0065] Granziera C., Wuerfel J., Barkhof F., Calabrese M., De Stefano N., Enzinger C. (2021). Quantitative magnetic resonance imaging towards clinical application in multiple sclerosis. Brain..

[b0070] Griswold M.A., Jakob P.M., Heidemann R.M., Nittka M., Jellus V., Wang J. (2002). Generalized autocalibrating partially parallel acquisitions (GRAPPA). Magn. Reson. Med..

[b0075] Hametner S., Endmayr V., Deistung A., Palmrich P., Prihoda M., Haimburger E. (2018). The influence of brain iron and myelin on magnetic susceptibility and effective transverse relaxation - A biochemical and histological validation study. Neuroimage..

[b0080] Helms G., Dechent P. (2009). Increased SNR and reduced distortions by averaging multiple gradient echo signals in 3D FLASH imaging of the human brain at 3T. J. Magn. Reson. Imaging.

[b0085] Helms G., Dathe H., Dechent P. (2008). Quantitative FLASH MRI at 3T using a rational approximation of the Ernst equation. Magn. Reson. Med..

[b0090] Helms G., Dathe H., Kallenberg K., Dechent P. (2008). High-resolution maps of magnetization transfer with inherent correction for RF inhomogeneity and T1 relaxation obtained from 3D FLASH MRI. Magn. Reson. Med..

[b0095] Helms G., Draganski B., Frackowiak R., Ashburner J., Weiskopf N. (2009). Improved segmentation of deep brain grey matter structures using magnetization transfer (MT) parameter maps. Neuroimage..

[b0100] Helms G., Dathe H., Dechent P. (2010). Modeling the influence of TR and excitation flip angle on the magnetization transfer ratio (MTR) in human brain obtained from 3D spoiled gradient echo MRI. Magn. Reson. Med..

[b0105] Jenkinson M., Beckmann C.F., Behrens T.E., Woolrich M.W., Smith S.M. (2012). Fsl. Neuroimage..

[b0110] Kaunzner U.W., Kang Y., Zhang S., Morris E., Yao Y., Pandya S. (2019). Quantitative susceptibility mapping identifies inflammation in a subset of chronic multiple sclerosis lesions. Brain..

[b0115] Kolb H., Absinta M., Beck E.S., Ha S.K., Song Y., Norato G. (2021). 7T MRI Differentiates Remyelinated from Demyelinated Multiple Sclerosis Lesions. Ann. Neurol..

[b0120] Koo T.K., Li M.Y. (2016). A Guideline of Selecting and Reporting Intraclass Correlation Coefficients for Reliability Research. J. Chiropr. Med..

[b0125] Kozlowski P., Rosicka P., Liu J., Yung A.C., Tetzlaff W. (2014). In vivo longitudinal Myelin Water Imaging in rat spinal cord following dorsal column transection injury. Magn. Reson. Imaging.

[b0130] Kutzelnigg A., Lucchinetti C.F., Stadelmann C., Bruck W., Rauschka H., Bergmann M. (2005). Cortical demyelination and diffuse white matter injury in multiple sclerosis. Brain..

[b0135] La Rosa F., Abdulkadir A., Fartaria M.J., Rahmanzadeh R., Lu P.J., Galbusera R. (2020). Multiple sclerosis cortical and WM lesion segmentation at 3T MRI: a deep learning method based on FLAIR and MP2RAGE. Neuroimage Clin..

[b0140] Lassmann H. (2018). Pathogenic Mechanisms Associated With Different Clinical Courses of Multiple Sclerosis. Front. Immunol..

[b0145] Laule C., Leung E., Lis D.K., Traboulsee A.L., Paty D.W., MacKay A.L. (2006). Myelin water imaging in multiple sclerosis: quantitative correlations with histopathology. Mult. Scler..

[b0150] Lee L.E., Ljungberg E., Shin D., Figley C.R., Vavasour I.M., Rauscher A. (2018). Inter-Vendor Reproducibility of Myelin Water Imaging Using a 3D Gradient and Spin Echo Sequence. Front. Neurosci..

[b0155] Levy S., Guertin M.C., Khatibi A., Mezer A., Martinu K., Chen J.I. (2018). Test-retest reliability of myelin imaging in the human spinal cord: Measurement errors versus region- and aging-induced variations. PLoS ONE.

[b0160] Lieury A., Chanal M., Androdias G., Reynolds R., Cavagna S., Giraudon P. (2014). Tissue remodeling in periplaque regions of multiple sclerosis spinal cord lesions. Glia..

[b0165] Lin P.Y., Chao T.C., Wu M.L. (2015). Quantitative susceptibility mapping of human brain at 3T: a multisite reproducibility study. AJNR Am. J. Neuroradiol..

[b0170] Liu T., Xu W., Spincemaille P., Avestimehr A.S., Wang Y. (2012). Accuracy of the morphology enabled dipole inversion (MEDI) algorithm for quantitative susceptibility mapping in MRI. IEEE Trans. Med. Imaging.

[b0175] Lublin F.D., Reingold S.C., Cohen J.A., Cutter G.R., Sorensen P.S., Thompson A.J. (2014). Defining the clinical course of multiple sclerosis: the 2013 revisions. Neurology..

[b0180] Lucchinetti C., Bruck W., Parisi J., Scheithauer B., Rodriguez M., Lassmann H. (2000). Heterogeneity of multiple sclerosis lesions: implications for the pathogenesis of demyelination. Ann. Neurol..

[b0185] Magliozzi R., Howell O., Vora A., Serafini B., Nicholas R., Puopolo M. (2007). Meningeal B-cell follicles in secondary progressive multiple sclerosis associate with early onset of disease and severe cortical pathology. Brain..

[b0190] Marques J.P., Kober T., Krueger G., van der Zwaag W., Van de Moortele P.F., Gruetter R. (2010). MP2RAGE, a self bias-field corrected sequence for improved segmentation and T1-mapping at high field. Neuroimage..

[b0195] Meyers S.M., Laule C., Vavasour I.M., Kolind S.H., Madler B., Tam R. (2009). Reproducibility of myelin water fraction analysis: a comparison of region of interest and voxel-based analysis methods. Magn. Reson. Imaging.

[b0200] Moccia M., van de Pavert S., Eshaghi A., Haider L., Pichat J., Yiannakas M. (2020). Pathologic correlates of the magnetization transfer ratio in multiple sclerosis. Neurology..

[b0205] Moore G.R., Leung E., MacKay A.L., Vavasour I.M., Whittall K.P., Cover K.S. (2000). A pathology-MRI study of the short-T2 component in formalin-fixed multiple sclerosis brain. Neurology..

[b0210] Mottershead J.P., Schmierer K., Clemence M., Thornton J.S., Scaravilli F., Barker G.J. (2003). High field MRI correlates of myelin content and axonal density in multiple sclerosis–a post-mortem study of the spinal cord. J. Neurol..

[b0215] Multiple L.H., Pathology S. (2018). Cold Spring Harb Perspect Med..

[b0220] Mustafi S.M., Harezlak J., Kodiweera C., Randolph J.S., Ford J.C., Wishart H.A. (2019). Detecting white matter alterations in multiple sclerosis using advanced diffusion magnetic resonance imaging. Neural Regen Res..

[b0225] Nguyen T.D., Wisnieff C., Cooper M.A., Kumar D., Raj A., Spincemaille P. (2012). T2 prep three-dimensional spiral imaging with efficient whole brain coverage for myelin water quantification at 1.5 tesla. Magn. Reson. Med..

[b0230] Nguyen T.D., Deh K., Monohan E., Pandya S., Spincemaille P., Raj A. (2016). Feasibility and reproducibility of whole brain myelin water mapping in 4 minutes using fast acquisition with spiral trajectory and adiabatic T2prep (FAST-T2) at 3T. Magn. Reson. Med..

[b0235] O'Muircheartaigh J., Vavasour I., Ljungberg E., Li D.K.B., Rauscher A., Levesque V. (2019). Quantitative neuroimaging measures of myelin in the healthy brain and in multiple sclerosis. Hum. Brain Mapp..

[b0240] Piredda G.F., Hilbert T., Thiran J.P., Kober T. (2021). Probing myelin content of the human brain with MRI: A review. Magn. Reson. Med..

[b0245] Rahmanzadeh R., Lu P.J., Barakovic M., Weigel M., Maggi P., Nguyen T.D. (2021). Myelin and axon pathology in multiple sclerosis assessed by myelin water and multi-shell diffusion imaging. Brain..

[b0250] Rahmanzadeh R., Lu P.J., Barakovic M., Weigel M., Maggi P., Nguyen T.D. (2021). Myelin and axon pathology in multiple sclerosis assessed by myelin water and multi-shell diffusion imaging. Brain.

[b0255] Rodriguez M., Scheithauer B.W., Forbes G., Kelly P.J. (1993). Oligodendrocyte injury is an early event in lesions of multiple sclerosis. Mayo Clin. Proc..

[b0260] Rodriguez M., Scheithauer B. (1994). Ultrastructure of multiple sclerosis. Ultrastruct. Pathol..

[b0265] Ropele S., Filippi M., Valsasina P., Korteweg T., Barkhof F., Tofts P.S. (2005). Assessment and correction of B1-induced errors in magnetization transfer ratio measurements. Magn. Reson. Med..

[b0270] Santin M.D., Didier M., Valabregue R., Yahia Cherif L., Garcia-Lorenzo D., Loureiro de Sousa P. (2017). Reproducibility of R2 * and quantitative susceptibility mapping (QSM) reconstruction methods in the basal ganglia of healthy subjects. NMR Biomed..

[b0275] Sati P., George I.C., Shea C.D., Gaitan M.I., Reich D.S. (2012). FLAIR*: a combined MR contrast technique for visualizing white matter lesions and parenchymal veins. Radiology.

[b0280] Schmierer K., Scaravilli F., Altmann D.R., Barker G.J., Miller D.H. (2004). Magnetization transfer ratio and myelin in postmortem multiple sclerosis brain. Ann. Neurol..

[b0285] Seewann A., Kooi E.J., Roosendaal S.D., Barkhof F., van der Valk P., Geurts J.J. (2009). Translating pathology in multiple sclerosis: the combination of postmortem imaging, histopathology and clinical findings. Acta Neurol. Scand..

[b0290] Sun D., Yu Z., Fang X., Liu M., Pu Y., Shao Q. (2017). LncRNA GAS5 inhibits microglial M2 polarization and exacerbates demyelination. EMBO Rep..

[b0295] Tabelow K., Balteau E., Ashburner J., Callaghan M.F., Draganski B., Helms G. (2019). hMRI - A toolbox for quantitative MRI in neuroscience and clinical research. Neuroimage..

[b0300] Thompson A.J., Banwell B.L., Barkhof F., Carroll W.M., Coetzee T., Comi G. (2018). Diagnosis of multiple sclerosis: 2017 revisions of the McDonald criteria. Lancet Neurol..

[b0305] van der Weijden C.W.J., Garcia D.V., Borra R.J.H., Thurner P., Meilof J.F., van Laar P.J. (2021). Myelin quantification with MRI: A systematic review of accuracy and reproducibility. Neuroimage..

[b0310] Vavasour I.M., Clark C.M., Li D.K., Mackay A.L. (2006). Reproducibility and reliability of MR measurements in white matter: clinical implications. Neuroimage..

[b0315] Vrenken H., Geurts J.J., Knol D.L., van Dijk L.N., Dattola V., Jasperse B. (2006). Whole-brain T1 mapping in multiple sclerosis: global changes of normal-appearing gray and white matter. Radiology.

[b0320] Vrenken H., Rombouts S.A., Pouwels P.J., Barkhof F. (2006). Voxel-based analysis of quantitative T1 maps demonstrates that multiple sclerosis acts throughout the normal-appearing white matter. AJNR Am. J. Neuroradiol..

[b0325] Weiskopf N., Suckling J., Williams G., Correia M.M., Inkster B., Tait R. (2013). Quantitative multi-parameter mapping of R1, PD(*), MT, and R2(*) at 3T: a multi-center validation. Front. Neurosci..

[b0330] Wicaksono K.P., Fushimi Y., Nakajima S., Yokota Y., Oshima S., Otani S. (2021). Two-Minute Quantitative Susceptibility Mapping From Three-Dimensional Echo-Planar Imaging: Accuracy, Reliability, and Detection Performance in Patients With Cerebral Microbleeds. Invest. Radiol..

[b0335] Wisnieff C., Ramanan S., Olesik J., Gauthier S., Wang Y., Pitt D. (2015). Quantitative susceptibility mapping (QSM) of white matter multiple sclerosis lesions: Interpreting positive susceptibility and the presence of iron. Magn. Reson. Med..

[b0350] Yaghmaie N., Syeda W.T., Wu C., Zhang Y., Zhang T.D., Burrows E.L. (2021). QSMART: Quantitative susceptibility mapping artifact reduction technique. Neuroimage..

